# Utilization and outcomes of nutrient-stimulated hormone therapies in patients undergoing bariatric surgery

**DOI:** 10.1038/s41366-026-02159-3

**Published:** 2026-07-20

**Authors:** Claudia R. Villatoro Santos, Hector D. Estrada Tock, Haoran Tang, Laura N. McEwen, Andrew Kraftson, William H. Herman, Amy E. Rothberg

**Affiliations:** 1 Division of Endocrinology, Metabolism & Lipid Research, Department of Medicine, Washington University School of Medicine, St. Louis, MO USA; 2 School of Information, University of Michigan, Ann Arbor, MI USA; 3 Department of Biostatistics, School of Public Health, University of Michigan, Ann Arbor, MI USA; 4 Division of Metabolism, Endocrinology, and Diabetes, Department of Internal Medicine, University of Michigan, Ann Arbor, MI USA; 5 Department of Epidemiology, School of Public Health, University of Michigan, Ann Arbor, MI USA

**Keywords:** Obesity, Epidemiology

## Abstract

**Objective:**

To describe the impact of nutrient-stimulated hormone (NuSH) therapies on weight loss by bariatric surgery type, diabetes status, and timing of initial NuSH prescription.

**Methods:**

Retrospective study of 1638 adults who underwent bariatric surgery at a single academic medical center between 2015 and 2022. Baseline demographics, NuSH therapy prescriptions, and follow-up weights were obtained from electronic health records. We calculated percent total weight loss (%TWL) over time using linear mixed models.

**Results:**

Patients who underwent Roux-en-Y gastric bypass (RYGB) had greater %TWL compared to those who underwent sleeve gastrectomy (SG). Those without diabetes had greater %TWL compared to those with diabetes. At 72 months, adjusted average %TWL was highest in patients who received NuSH prescriptions both before and after surgery (22.7%, 95% CI: 17.9–27.5), followed by those with prescriptions only after surgery (19.9%, 95% CI: 18.3–21.4) and those without prescriptions (19.6%, 95% CI: 18.1–21.2). The greatest %TWL was seen in patients who had prescriptions within 6 months after surgery.

**Conclusion:**

Individuals who underwent RYGB and who did not have diabetes had the highest %TWL. Receiving a NuSH prescription both before and after surgery and early NuSH initiation were associated with the greatest %TWL.

## Introduction

In 2022, approximately 280,000 bariatric surgery procedures were performed in the United States. Of these, sleeve gastrectomy (SG) and Roux-en-Y gastric bypass (RYGB) were the most commonly performed, representing 57% and 22% of the total, respectively [1]. Bariatric surgery is currently the most effective treatment for severe obesity. A meta-analysis of randomized trials and observational studies reported percent total weight loss (%TWL) ranging from 17 to 28% in the short term (1–2 years) and 13 to 25% in the long-term (3–5 years) [2]. Longer-term follow-up studies have reported maintenance of weight loss for up to 12 years [2, 3]. Bariatric surgery is also a very effective treatment for type 2 diabetes in patients with severe obesity. A pooled analysis of randomized trials found that glycemic control was superior in patients with type 2 diabetes who underwent bariatric surgery compared to those who received medical management for up to 12 years [4]. RYGB was associated with better glycemic outcomes when compared to SG and adjustable gastric banding [4]. At the study location, SG is the preferred surgical option due to shorter operative time, decreased length of stay, and fewer long-term postoperative management requirements [4, 5].

Despite its effectiveness, weight regain is now being reported more frequently following bariatric surgery, especially after SG [6]. Because the frequency of SG is increasing and that of RYGB is decreasing, we anticipate that weight regain will become an increasingly common problem in clinical practice and will require effective treatment strategies.

Currently, the literature provides no standard definition of weight regain. Several definitions have been proposed and estimates of weight regain vary depending on the definition used. One definition used in a recent systematic review was the median percentage of maximum weight lost regained [7]. It was reported to range from 19 to 26% [7].

Current treatment strategies for weight regain include interventions to increase dietary and physical activity adherence, anti-obesity medications (AOMs), and surgical revisions. Several observational studies have evaluated the use of glucagon-like peptide-1 receptor agonists (GLP-1 RAs) for the treatment of both suboptimal clinical responses and weight regain after bariatric surgery. The most common AOM studied was liraglutide as this was the first GLP-1 RA approved by the Food and Drug Administration (FDA) for the treatment of obesity in December 2014 [8–11]. In June 2021, semaglutide was approved by the FDA for the treatment of obesity. The dual gastric inhibitory polypeptide (GIP) and glucagon-like peptide-1 (GLP-1) receptor agonist tirzepatide was not approved by the FDA for the treatment of obesity until November 2023. The term “nutrient-stimulated hormone” (NuSH) therapy has been used to refer to these classes of medications [12].

In this study, we evaluated the effectiveness of bariatric surgery for weight loss and described the current use of NuSH therapies and their impact on weight loss by the type of surgery performed, diabetes status, and the period during which NuSH therapy was prescribed. Our goal was to better understand the utilization of NuSH therapies in patients with obesity undergoing RYGB and SG with and without diabetes to inform how NuSH therapies might be most effectively used and to highlight the cost and potential cost-effectiveness of alternative approaches to surgical and medical treatment.

## Methods

### Study population

We performed a retrospective cohort study of all adult patients 18–65 years of age who underwent RYGB and SG between 01/01/2015 and 12/31/2022 at a single large academic medical center. The date 01/01/2015 was selected to correspond with the date of FDA approval of liraglutide for the treatment of obesity (12/23/2014). Conversions from SG to RYGB (*n* = 12) were classified as SG and all weight values after the date of the second surgery were censored. Patients who had revision surgeries during the study period, but initial bariatric procedures performed before 01/01/2015, and patients who were diagnosed with malignancies or pregnancies during the study period were excluded from the study.

### Ethics approval and consent to participate

The study protocol was reviewed and approved by the University of Michigan Institutional Review Board (IRB), study ID HUM00245147. The IRB granted a waiver of informed consent, as the research involved retrospective secondary analyses of a HIPAA-compliant limited dataset.

### Data collection

Data were collected using the health system’s clinical data warehouse, a web-based software program, and chart reviews. The datasets generated during and/or analyzed during the current study are available from the corresponding author on reasonable request. Surgical procedure types and dates were identified using Current Procedural Terminology (CPT) codes, International Classification of Diseases (ICD-9) and procedural ICD-10 codes (based on the Centers for Medicare & Medicaid Services (CMS) reimbursement scheme). We collected demographic information including age, sex, race, and insurance type. We collected anthropometric data including height, weight, blood pressure, and Body Mass Index (BMI) values over time. We used each patient’s residential zip code to perform geocoding to assess median neighborhood income, percent unemployment, percent receiving Supplemental Nutrition Assistance Program (SNAP) benefits, and percent education level classified as less than high school diploma, high school and/or some college, and bachelor’s degree or higher. We collected information on medical history and the dates of NuSH therapy prescriptions for the GLP-1 RAs liraglutide, semaglutide, dulaglutide, and exenatide, and the dual GIP and GLP-1 agonist tirzepatide. There were no patients who had prescriptions for lixisenatide or albiglutide. We collected baseline laboratory data on platelet, lipid, hemoglobin A1C, serum creatinine, lipase, aspartate transaminase (AST), and alanine transaminase (ALT) levels, estimated glomerular filtration rate (eGFR), and urine albumin-creatinine ratio. Laboratory data were obtained from the health system’s data warehouse and included tests ordered and performed within the system. However, the dataset also included laboratory results from external facilities that were incorporated into patients’ electronic health records. Prescription data were derived from prescription orders generated by providers practicing within the health system.

### NuSH therapy prescriptions

Patients were classified as receiving NuSH therapy if they had at least one prescription during the study period. The length of each prescription period was calculated as the difference between the prescription period’s stop date and its corresponding start date. The cumulative exposure to NuSH therapy was calculated as the sum of all prescription periods, in days, with the result expressed as months. For the analysis of the association between NuSH therapy and weight loss, patients were categorized as never having received a NuSH prescription, receiving a prescription only before surgery, before and after surgery, or only after surgery.

### Percent total weight loss (%TWL)

To evaluate the effects of surgery on weight, we defined the baseline weight as the weight at the time of surgery. Follow-up weights were assessed from the electronic health record every 3 months for up to 72 months after surgery. The weight used at each time point was the last weight recorded within the 3-month interval. The number of follow-up measurements varied among individuals, with a minimum of 0 and maximum of 24 reported. The median was 7 and the interquartile range was 9. Missing values were not imputed. Only available weights were used to calculate %TWL. %TWL associated with bariatric surgery was calculated as the difference between the baseline weight and the weight during each interval divided by the baseline weight multiplied by 100.

To evaluate the effect of NuSH therapy on %TWL, we redefined the baseline weight as the weight on the date of the first NuSH therapy prescription following surgery. The follow-up weights were assessed every 3 months for up to 45 months. For those who did not receive a NuSH prescription after surgery (never and before surgery only groups), there was no date of first prescription after surgery to use as an index date, so an equivalent index date was calculated. First, an average time to first prescription was calculated among those who had a prescription after surgery, grouped by type of surgery and diabetes status (“Average months to prescription”, Table S2). This time to first prescription was added to the surgery date for each patient without a NuSH prescription after surgery, according to their type of surgery and diabetes status, to obtain an equivalent index date. The definition of %TWL following the date of the first NuSH therapy prescription or the index date was the same as used in the analysis from date of surgery.

### Data analysis

Descriptive statistics were used to characterize the study population, %TWL after surgery and after initiation of NuSH therapy, and prescription practices for NuSH therapy. Continuous variables were reported as means and standard deviations or medians and interquartile ranges, and categorical variables were summarized as frequency distributions. We compared the characteristics of patients who were and were not prescribed NuSH therapy by surgery type using t-tests for continuous variables and chi-square tests for categorical variables.

We evaluated unadjusted trends in %TWL over time by NuSH therapy and NuSH therapy timing, stratified by surgery type and diabetes status.

To assess the impact of surgery and NuSH therapy on %TWL independent of age, sex, baseline BMI, surgery type, and diabetes status, we performed multivariate analyses. Specifically, we used linear mixed models to evaluate the effect of surgery and the association between NuSH therapy prescriptions and %TWL accounting for the correlation of repeated measures of a patient’s weight over time. We used marginal models without a random intercept as there was no significant variability explained in the model by the random intercept. We used an autoregressive covariance structure as this best fit the data. All models were adjusted for age, sex, BMI on the date of surgery or the date of the first NuSH therapy prescription, baseline hemoglobin A1C, diabetes status, surgery type, hypertension, and percent of people with a high school diploma or some college in the patient’s residential zip code. We included an interaction term between NuSH therapy prescription and time to assess trends over time for %TWL. Time was introduced in the model as a categorical variable to account for non-linear trends in weights over time. We obtained least-squares means as population averages of %TWL over time from the adjusted model.

We also evaluated if the time to initial NuSH therapy prescription had a differential impact on %TWL for those who received a prescription after surgery (excluding those who never received a prescription and those who received a prescription only before surgery) and ran adjusted linear mixed models and classified date of NuSH therapy prescription between 0 and 6 months, 7 and 12 months, 13 and 24 months, and more than 24 months after surgery.

## Results

We identified 1638 eligible patients. The average age (±SD) was 42.4 (±10.0) years. Women accounted for 80% of the population and 79% were white. There were 1461 (89%) patients who underwent SG and 177 (11%) who underwent RYGB. Five hundred fifty-six (34%) patients received prescriptions for NuSH therapy. Among the patients who underwent SG, 36% (*n* = 520) received prescriptions for NuSH therapy. In contrast, only 20% (*n* = 36) of the patients in the RYGB group ever received prescriptions for NuSH therapy. Therefore, of the 556 patients who received prescriptions, 520 (94%) belonged to the SG group and 36 (6%) belonged to the RYGB group. Baseline BMI at the time of surgery was significantly higher among those who underwent SG compared to those who underwent RYGB (46.5 ± 7.0 vs 44.6 ± 7.2 kg/m^2^, *p* = 0.001). Those who received prescriptions and underwent SG had significantly lower baseline BMIs, and those who received prescriptions and underwent RYGB had a significantly higher baseline BMIs than those who never received NuSH therapy prescriptions (Tables 1 and S1).

**Table 1:** Summary of baseline characteristics of the study population by sleeve gastrectomy (SG) or Roux-en-Y gastric bypass (RYGB) and treatment groups.

Characteristic	Total *N* = 1638	SG *N* = 1461 (89%)	RYGB *N* = 177 (11%)	*p*-value^a^	SG			RYGB		
					NuSH *N* = 520 (36%)	Non-NuSH *N* = 941 (64%)	*p*-value^a^	NuSH *N* = 36 (20%)	Non-NuSH *N* = 141 (71%)	*p*-value^a^
Age	42.4 ± 10.0	42.5 ± 10.0	41.2 ± 10.0	0.11	44.5 ± 9.2	41.4 ± 10.2	<0.0001	43.7 ± 9.6	40.6 ± 10.1	0.10
Female, *n* (%)	1318 (80)	1167 (80)	151 (85)	0.09	424 (82)	743 (79)	0.24	32 (89)	119 (84)	0.61
Race, *n* (%)				0.04			0.09			0.44
White	1291 (79)	1139 (78)	152 (86)		389 (75)	750 (80)		29 (81)	123 (87)	
African American	269 (16)	251 (17)	18 (10)		103 (20)	148 (16)		5 (14)	13 (9)	
Other	78 (5)	71 (5)	7 (4)		28 (5)	43 (5)		2 (6)	5 (4)	
Insurance type, *n* (%)				0.99			0.39			0.28
Medicaid/Medicare	435 (27)	388 (27)	47 (27)		145 (28)	243 (26)		7 (19)	40 (28)	
Private	1203 (73)	1073 (73)	130 (73)		375 (72)	698 (74)		29 (81)	101 (72)	
Baseline BMI, kg/m^2^	46.3 ± 7.0	46.5 ± 7.0	44.6 ± 7.2	0.001	46.0 ± 6.9	46.8 ± 7.0	0.04	47.5 ± 5.7	43.9 ± 7.4	0.007
Diabetes, *n* (%)	523 (32)	478 (33)	45 (25)	0.05	239 (46)	239 (25)	<0.0001	13 (36)	32 (23)	0.10

Continuous data reported as mean ± SD or median (IQR).

^a^*p*-value from *χ*^2^ tests for categorical variables and t-test for continuous variables.

There were 502 (31%) patients with diabetes in the study population. Of those, 459 (91%) underwent SG and 43 (9%) underwent RYGB. The proportion of patients with diabetes who received NuSH therapy prescriptions was higher compared to those without diabetes (48% vs 27%, respectively). We did not have information as to whether the medications were prescribed for diabetes or weight management. The pattern of use was similar in each procedure group (Table 1).

### Prescription practices

**Fig. 1:**
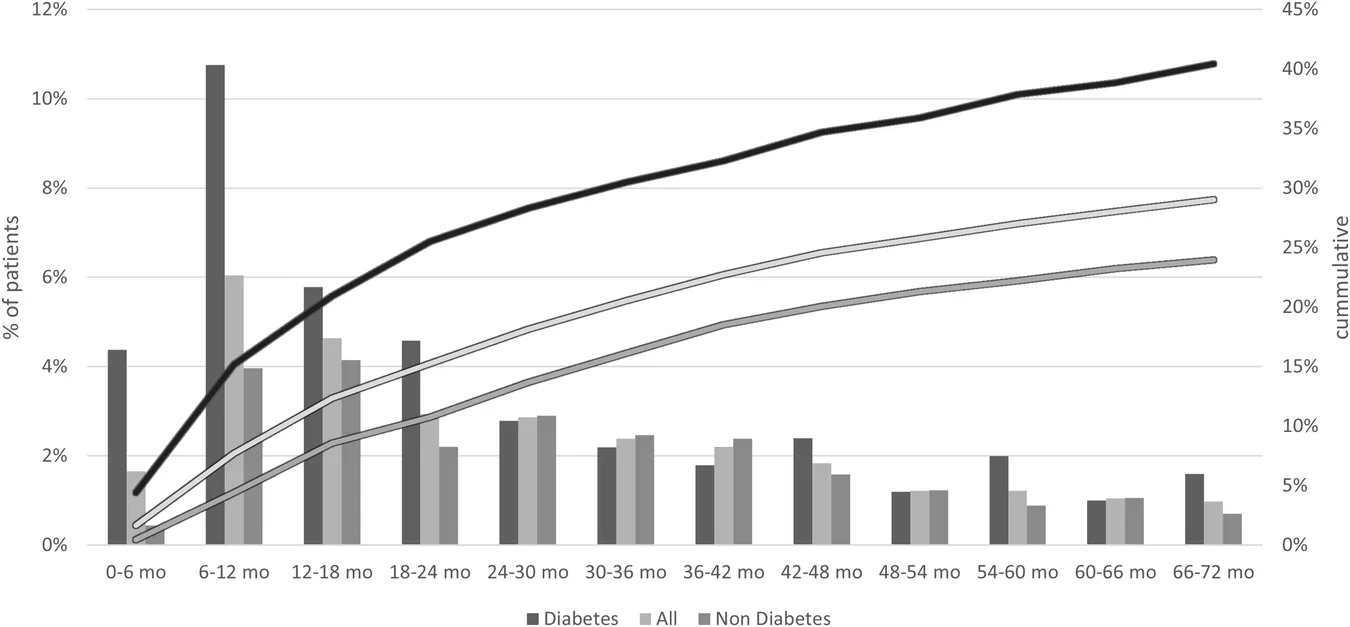
Cumulative percentage of patients prescribed NuSH therapy after surgery by diabetes status. Lines indicate cumulative incidence.

The percentage of patients who received NuSH therapy prescriptions was highest between 6 and 12 months after surgery and slowly declined thereafter. Patients with diabetes were more likely to be prescribed NuSH therapy compared to those without diabetes except between 24 and 42 months, when those without diabetes were more likely to receive prescriptions (Fig. 1). The average time to the first NuSH therapy prescription after surgery was 30 months. Patients who underwent RYBG and had diabetes had the shortest time to NuSH therapy prescription whereas those who underwent SG and did not have diabetes had the longest time to NuSH therapy prescription (Table S2).

### Surgery type and %TWL

**Fig. 2:**
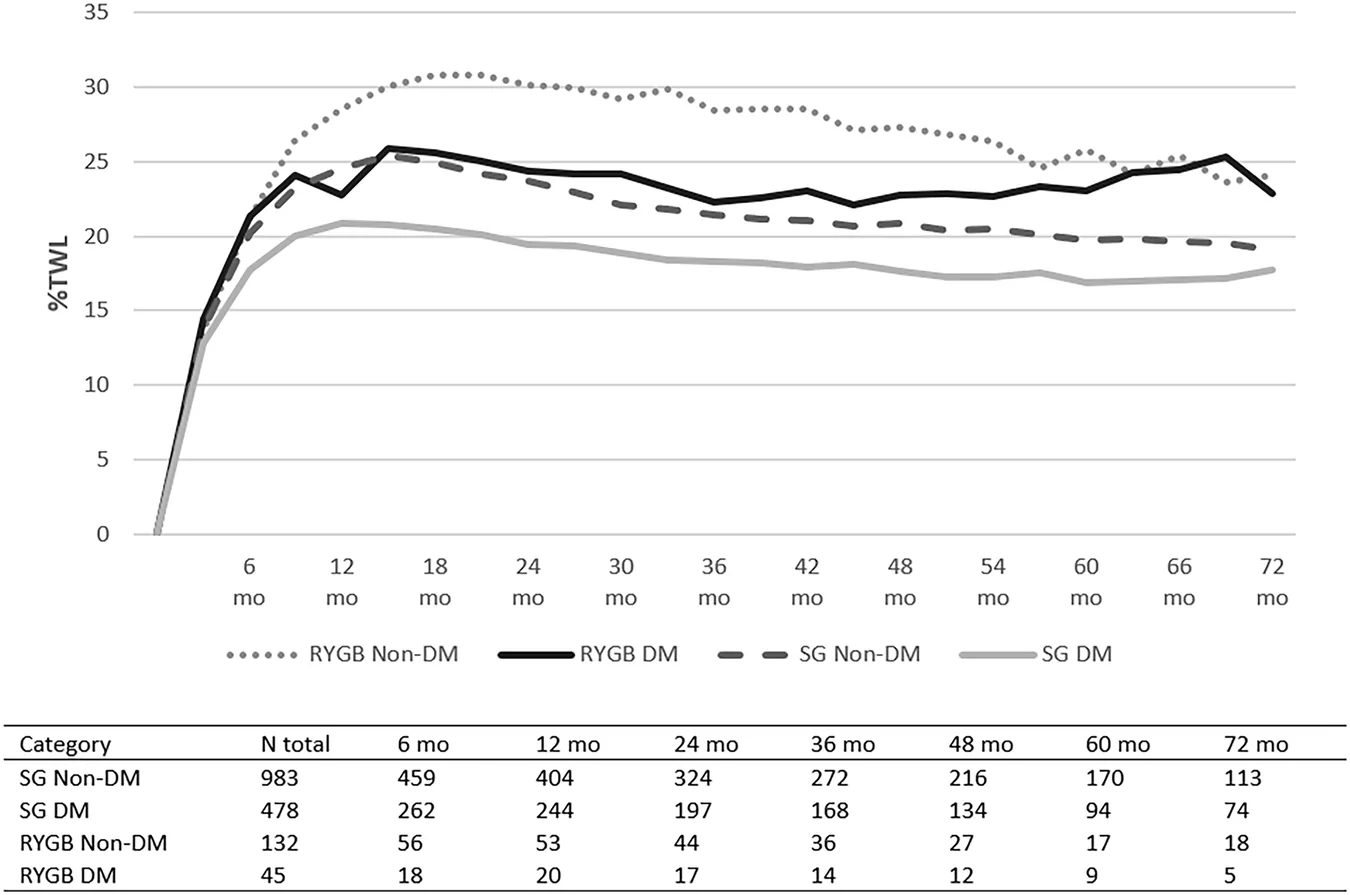
Unadjusted average %TWL^1^ by sleeve gastrectomy or Roux-en-Y gastric bypass and diabetes status, from date of surgery. ^1^Table represents the number of participants at each time point and overall percentage of patients with diabetes within each category.

Starting from the date of surgery, unadjusted %TWL was highest at 1–2 years for all patients. Those in the RYBG group had significantly higher %TWL over time compared to those in the SG group. After stratification by surgery type and diabetes status, those in the RYGB group and without diabetes had the highest %TWL followed by those in the RYGB group with diabetes. Those without diabetes in the SG group had higher %TWL than those with diabetes, but lower than those in the RYBG group (Fig. 2). Those with diabetes in the SG group had the lowest %TWL.

### NuSH therapy prescription and %TWL

**Fig. 3:**
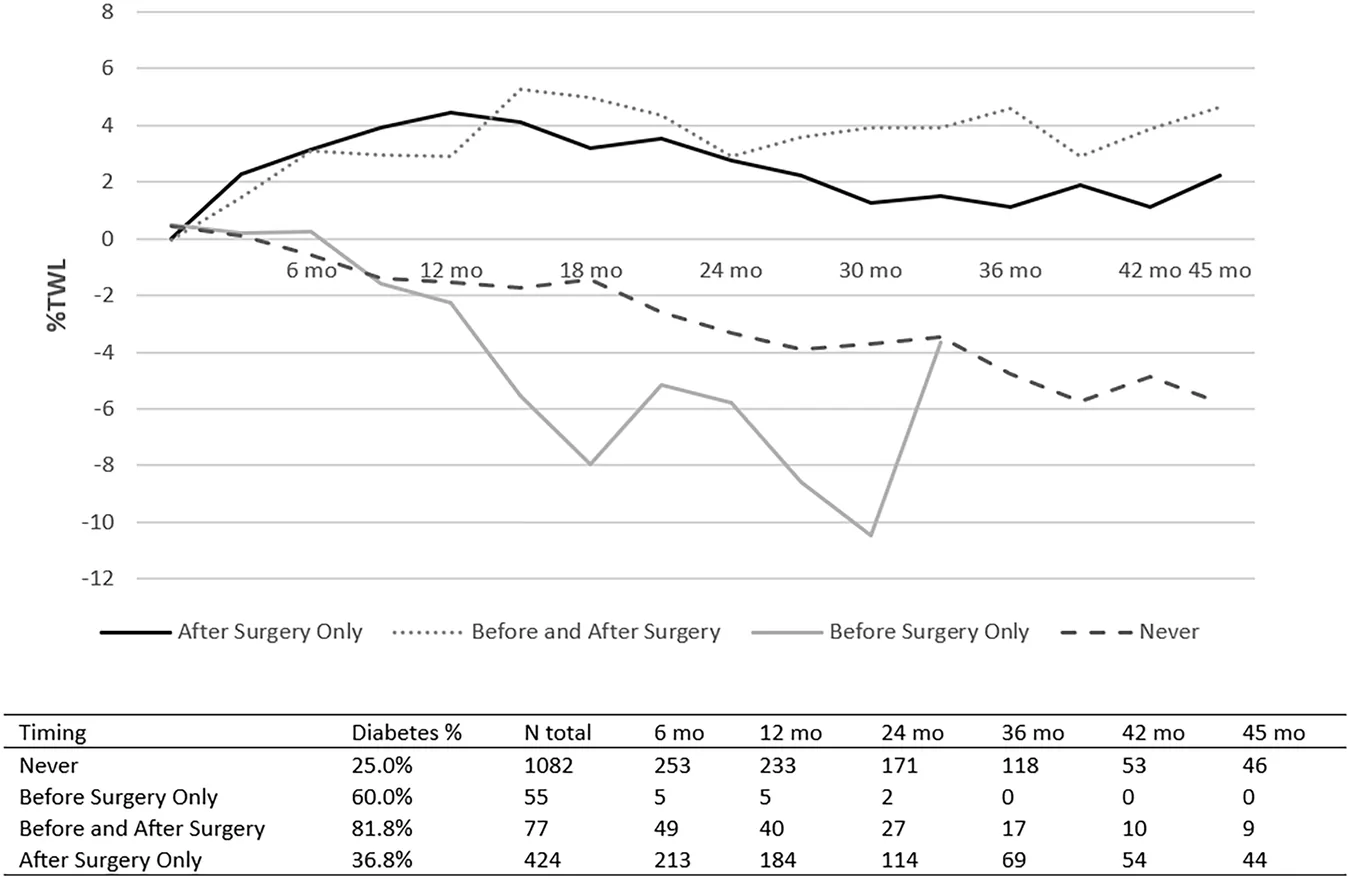
Adjusted^1^ average %TWL^2,3^ from date of first NuSH therapy prescription by prescription timing. ^1^Table represents the number of participants at each time point and overall percentage of patients with diabetes within each category. ^2^Model was adjusted for age, sex, BMI at first prescription, diabetes status, baseline hemoglobin A1C, type of surgery, hypertension, and percent of persons in zip code with high school diploma and/or some college. ^3^Estimated from least squared means of the interaction term between treatment and time from adjusted model. Data were truncated at 45 months due to paucity of follow up information after that time point.

Starting from the date of first NuSH therapy prescription, or the index date for those who were not prescribed NuSH therapy after surgery, those who received NuSH therapy prescriptions before and after surgery and only after surgery had the highest adjusted average %TWL over time. At 12 months, those with prescriptions before and after surgery lost on average, 3% more of their total weight since the initial prescription (95% CI: 0.7%, 5.0%) compared to those who did not receive NuSH therapy. At 12 months, those who received prescriptions only after surgery lost an average of 4.5% more of their total body weight after the initial prescription (95% CI: 3.3%, 5.6%) compared to those who did not receive NuSH therapy. At 45 months, the adjusted average %TWL was highest for those who received NuSH prescriptions before and after surgery, followed by the group that received prescriptions only after surgery (4.6% (95% CI: 0.2%, 9.0%) vs 2.2% (95% CI: 0.2%, 4.2%), respectively). The groups of patients who never received prescriptions for NuSH therapy or received prescriptions only before surgery demonstrated gradual weight regain after surgery. (Fig. 3 and Table S3).

**Fig. 4:**
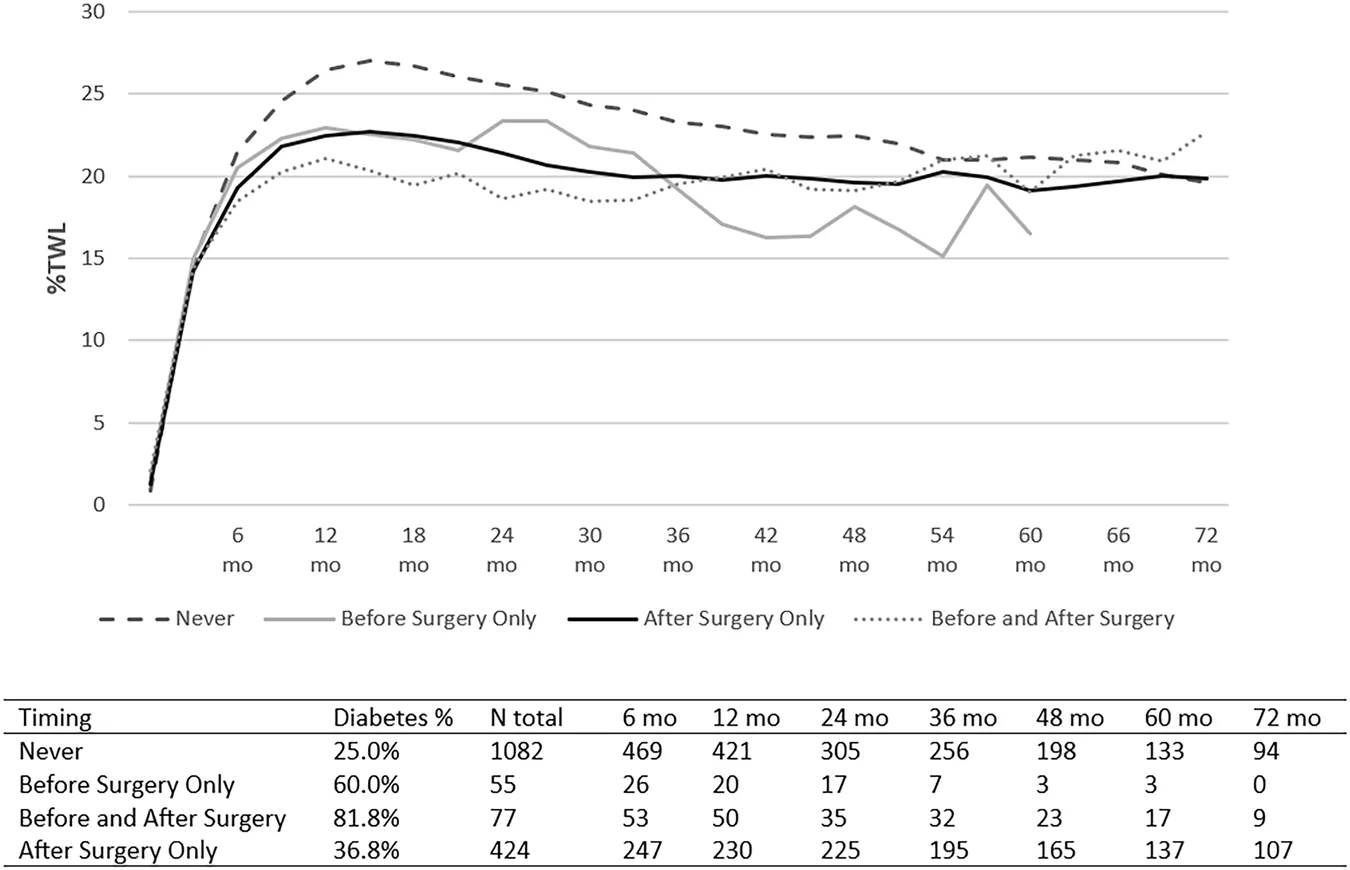
Adjusted^1^ average %TWL^2,3^ from date of surgery by prescription timing. ^1^Table represents the number of participants at each time point and overall percentage of patients with diabetes within each category. ^2^Model was adjusted for age, sex, baseline BMI, diabetes status, baseline hemoglobin A1C, type of surgery, hypertension, and percent of persons in zip code with high school diploma and/or some college. ^3^Estimated from least squared means of the interaction term between treatment and time from adjusted model.

Starting from the date of surgery (Fig. 4), those who never received NuSH therapy prescriptions achieved the greatest adjusted %TWL up to 54 months of follow up. The groups that received prescriptions before and after surgery and only after surgery achieved modest increases in adjusted %TWL after the average time to initial prescription (30 months), closing the gap in %TWL with the group that never received NuSH therapy prescriptions at 54 months. At 72 months, the adjusted average %TWL was 19.6% (95% CI: 18.1%, 21.2%) for those who never received prescriptions, 22.7% (95% CI: 17.9%, 27.5%) for those who received prescriptions before and after surgery, and 19.9% (95% CI: 18.3%, 21.4%) for those who received prescriptions only after surgery. Patients who received NuSH therapy prescriptions only before surgery had a lower adjusted %TWL compared to those who did not receive prescriptions before surgery (never and after surgery only) and had the smallest adjusted %TWL over time (Fig. 4 and Table S4).

**Fig. 5:**
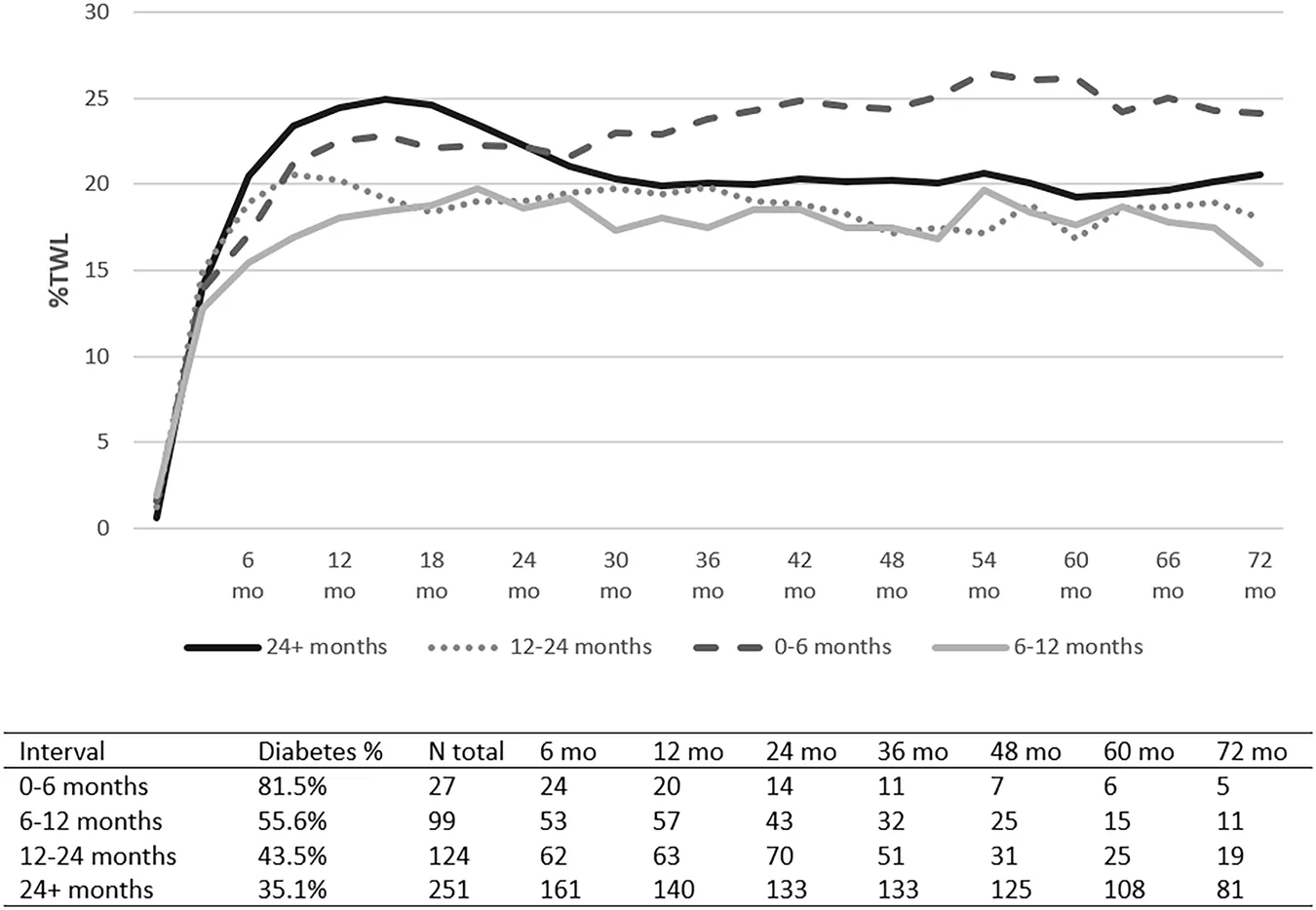
Adjusted^1^ average %TWL^2,3^ from date of surgery by first prescription time interval^4^ for those who received a NuSH therapy prescription after surgery^5^. ^1^Table represents the number of participants at each time point and overall percentage of patients with diabetes within each category. ^2^Model was adjusted for age, sex, baseline BMI, diabetes status, baseline hemoglobin A1C, type of surgery, hypertension, and percent of persons in zip code with high school diploma and/or some college. ^3^Estimated from least squared means of the interaction term between treatment and time from adjusted model. ^4^Time intervals indicate the period in which patients received their first NuSH therapy prescription after surgery. ^5^Includes only patients who received a NuSH therapy prescription before and after surgery or only after surgery.

We also examined the effect of the time to the initial NuSH therapy prescription for those who received a prescription after surgery (excluding those who never received prescriptions and those who received prescriptions only before surgery). Patients who received their first prescription within the first 6 months after surgery achieved the highest %TWL at 72 months (23.6% (95% CI: 16.1%, 30.8%)). Those prescribed NuSH therapy after 24 months after surgery achieved the second largest %TWL at 72 months (20.2% (95% CI: 18.1%, 22.3%)). Those who were prescribed NuSH therapy 6–12 months after surgery had the lowest %TWL at 72 months (14.7% (95% CI: 10.1%, 19.4%)) (Fig. 5 and Table S5).

## Discussion

Obesity is a chronic, relapsing condition that requires long-term, multi-modal treatment. Bariatric surgery is currently the most effective treatment for this condition but weight regain remains a concern despite initial treatment effectiveness. Use of obesity modifying medications like NuSH therapy is becoming an increasingly prevalent strategy to address weight regain following surgical therapy.

In this retrospective cohort study of patients who underwent bariatric surgery at a single large academic medical center, we found that more than one-third (34%) received NuSH therapy prescriptions. The rate we observed is higher than the reported use of liraglutide and semaglutide in another post-bariatric surgery population, most likely due to the more recent time period of our study and the approval and more frequent use of effective NuSH therapies for weight loss [13].

In our population, patients with and without diabetes who underwent RYGB had higher %TWL compared to those who underwent SG. The %TWL was lower among patients with diabetes compared to those without diabetes undergoing the same type of bariatric procedure. These findings are consistent with the literature [14, 15] and suggest that RYGB might improve weight loss outcomes compared to SG, especially in patients with diabetes.

It is important to understand the associations among surgical strategies, patient selection, and timing of NuSH therapy prescriptions and subsequent outcomes. We found that patients who underwent SG were more likely to be prescribed NuSH therapy than those who underwent RYGB. The cost of NuSH therapy is high in the US with list prices for semaglutide, dulaglutide and tirzepatide more than $1000 per month [15, 16]. Self-pay prices for semaglutide (Wegovy) and tirzepatide (Zepbound) available through manufacturers’ pharmacies range from $350 to $500 per month [17, 18]. In the US, list21 price reflects the manufacturer’s published price before any discounts or rebates. Self-pay prices indicate the prices paid by patients without insurance, which are typically lower than list prices. Considering the high costs of the NuSH therapy, RYBG may be more cost-effective than SG over the long-term, further supporting the notion that RYBG may be the bariatric surgery procedure of choice, especially for patients with diabetes.

Interestingly, those who never received a NuSH therapy prescription achieved the greatest adjusted %TWL for up to 54 months following surgery. At 72 months, the adjusted average %TWL was 19.6% for those who never received a NuSH therapy prescription, 22.7% for those who received a prescription before and after surgery, and 19.9% for those who received a prescription after surgery only. We hypothesize that these findings may in part be explained by confounding by indication which occurs when the indication for treatment is associated with the outcome [19]. In the present case, clinicians may have been more likely to prescribe NuSH therapy for patients who had lower %TWL over time. It is difficult to adjust for confounding by indication. However, we attempted to minimize the impact of confounding by indication by describing %TWL over time and evaluating the association between NuSH therapy and %TWL starting on the date of initial prescription. As might be expected, we saw a progressive increase in %TWL with NuSH therapy after 54 weeks. A recent case-control study showed that preoperative use of semaglutide did not result in higher %TWL which is not consistent with our findings [20], likely related to differences in study design and inclusion of only one type of medication.

In our analysis of %TWL after initiation of NuSH therapy, we found that patients who received NuSH therapy prescriptions after surgery had greater %TWL compared to patients who never received prescriptions or who received prescriptions only before surgery. At 45 months after initial prescription, the average adjusted %TWL was 4.6% in the group that received NuSH therapy before and after surgery and 2.2% in the group that received therapy only after surgery, whereas the group of patients who never received NuSH therapy had an average weight increase of 5.8% over the same timeframe. Several retrospective studies described %TWL ranging from 5.3 to 13.4% after liraglutide treatment for insufficient weight loss or weight regain following bariatric surgery [21]. Estimates for weight loss are higher for semaglutide compared to liraglutide. Our estimates are similar but not entirely comparable to those from previous retrospective studies, as we were not able to distinguish between types of NuSH therapies, potential differences in time to administration after bariatric surgery, and population characteristics. It is important to highlight that, based on the results for those who received prescriptions before and after surgery, patients may benefit from using NuSH therapy in preparation for surgery to optimize their outcomes and as adjunctive therapy after surgery in cases of suboptimal clinical response or weight regain.

The biological mechanism by which NuSH therapy prevents weight regain after bariatric surgery appears to be related to increasing GLP-1 levels. After bariatric surgery, the postprandial levels of GLP-1 increase by as early as 3 days. The duration of the effect depends on the type of surgical procedure. However, the levels of fasting GLP-1 remain lower after surgery in people with obesity than in lean controls. It has been hypothesized that NuSH therapy prevents weight regain after bariatric surgery by increasing fasting levels of GLP-1 and maintaining the increases in postprandial GLP-1 levels that tend to decrease over time following surgery [21].

We also found that %TWL was different based on the timing of initiation of NuSH therapy. Those who received a prescription within 6 months of surgery had the highest %TWL over time. Previous studies have demonstrated a wide range of time to initiation of NuSH therapy and therefore, we cannot directly compare our results or determine if there is an optimal time to initiate these medications after surgery.

Our study is not without limitations. Although we had information about prescription fills for NuSH therapy, we could not evaluate actual medication use or dosages. Due to the retrospective nature of our study and the use of diagnostic codes, there could also have been issues with the assignment of surgery type. We minimized this by adjudicating surgical status using individual chart reviews. The observational nature of the study precludes causal inference and unmeasured confounding is still possible. We were, however, able to adjust our estimates of %TWL for commonly reported confounders. Our population primarily included white women and may not be representative of the general population. The characteristics of our population were, however, similar to other previously described populations that undergo bariatric surgery. Due to the small sample size, we could not evaluate associations with individual medications.

Our study has several advantages. We evaluated a large population of patients undergoing bariatric surgery at a single large academic center and were able to have approximately 6 years of follow up to evaluate %TWL over time.

We found that RYGB results in a greater %TWL than SG and that individuals with diabetes achieve a lower %TWL than those without diabetes, regardless of the procedure performed. NuSH therapy prescriptions are more frequent for patients who undergo SG compared to those who undergo RYGB and for patients with diabetes compared to those without diabetes. Receiving a prescription after surgery leads to a higher %TWL compared to not receiving a prescription. We recommend RYBG as the bariatric procedure of choice, particularly for patients with diabetes, and hypothesize RYGB may be more cost-effective than SG after accounting for the current utilization and price of NuSH therapies. More studies are needed to elucidate the optimal time to initiate NuSH therapy in preparation for or as an adjunct to bariatric surgery and the cost-effectiveness of RYBG vs SG after accounting for the use of NuSH therapies. This information should be used to create guidelines that address pre/post operative use of NuSH therapies and indications for implementation.

## Supplementary information

Supplementary material

## Data Availability

The datasets generated during and/or analyzed during the current study are available from the corresponding author on reasonable request.

## References

[1] Clapp B, Ponce J, Corbett J, Ghanem OM, Kurian M, Rogers AM, et al. American Society for Metabolic and Bariatric Surgery 2022 estimate of metabolic and bariatric procedures performed in the United States. Surg Obes Relat Dis. 2024;20:425–31. 10.1016/j.soard.2024.01.012 38448343

[2] Kim JC, Kim M-G, Park JK, Lee S, Kim J, Cho YS, et al. Outcomes and adverse events after bariatric surgery: an updated systematic review and meta-analysis, 2013–2023. J Metab Bariatr Surg. 2023;12:76–88. 10.17476/jmbs.2023.12.2.76 PMC10771970 38196785

[3] Adams TD, Davidson LE, Litwin SE, Kim J, Kolotkin RL, Nanjee MN, et al. Weight and metabolic outcomes 12 years after gastric bypass. N Engl J Med. 2017;377:1143–55. 10.1056/NEJMoa1700459 PMC5737957 28930514

[4] Courcoulas AP, Patti ME, Hu B, Arterburn DE, Simonson DC, Gourash WF, et al. Long-term outcomes of medical management vs bariatric surgery in type 2 diabetes. JAMA. 2024;331:654–64. 10.1001/jama.2024.0318 PMC10900968 38411644

[5] Bregion PB, Reis AM, Jucá RH, de Oliveira-Filho JR, da Rocha Soares GA, Cazzo E, et al. Patients with severe obesity undergoing Roux-En-Y gastric bypass versus sleeve gastrectomy: a systematic review and an updated meta-analysis. Obes Surg. 2025;35:1146–59. 10.1007/s11695-025-07743-6 39964665

[6] Akpinar EO, Liem RSL, Nienhuijs SW, Greve JWM, Marang-van de Mheen PJ. Dutch Audit for treatment of obesity research group. weight recurrence after sleeve gastrectomy versus Roux-en-Y gastric bypass: a propensity score matched nationwide analysis. Surg Endosc. 2023;37:4351–9. 10.1007/s00464-022-09785-8 PMC10234854 36745232

[7] King WC, Hinerman AS, Courcoulas AP. Weight regain after bariatric surgery: a systematic literature review and comparison across studies using a large reference sample. Surg Obes Relat Dis. 2020;16:1133–44. 10.1016/j.soard.2020.03.034 32446593

[8] Muratori F, Vignati F, Di Sacco G, Gavazzi L, Pellegrino D, Del Prete M. Efficacy of liraglutide 3.0 mg treatment on weight loss in patients with weight regain after bariatric surgery. Eat Weight Disord. 2022;27:2775–81. 10.1007/s40519-022-01403-9 PMC9556416 35763245

[9] Rye P, Modi R, Cawsey S, Sharma AM. Efficacy of high-dose liraglutide as an adjunct for weight loss in patients with prior bariatric surgery. Obes Surg. 2018;28:3553–8. 10.1007/s11695-018-3393-7 30022424

[10] Wharton S, Kuk JL, Luszczynski M, Kamran E, Christensen RAG. Liraglutide 3.0 mg for the management of insufficient weight loss or excessive weight regain post-bariatric surgery. Clin Obes. 2019;9:e12323. 10.1111/cob.12323 PMC6771702 31183988

[11] Rubio MA, Ramos-Leví AM. Initial experience with alternate-day liraglutide for weight regain following bariatric surgery. Obes Surg. 2021;31:4216–8. 10.1007/s11695-021-05535-2 34212346

[12] Jastreboff AM, Kushner RF. New frontiers in obesity treatment: GLP-1 and nascent nutrient-stimulated hormone-based therapeutics. Annu Rev Med. 2023;74:125–39. 10.1146/annurev-med-043021-014919 36706749

[13] Firkins SA, Chittajallu V, Flora B, Yoo H, Simons-Linares R. Utilization of anti-obesity medications after bariatric surgery: analysis of a large national database. Obes Surg. 2024;34:1415–24. 10.1007/s11695-024-07181-w 38512645

[14] Iepsen EW, Torekov SS, Holst JJ. Liraglutide for Type 2 diabetes and obesity: a 2015 update. Expert Rev Cardiovasc Ther. 2015;13:753–67. 10.1586/14779072.2015.1054810 26106933

[15] Bergmann NC, Davies MJ, Lingvay I, Knop FK. Semaglutide for the treatment of overweight and obesity: a review. Diabetes Obes Metab. 2023;25:18–35. 10.1111/dom.14863 PMC10092086 36254579

[16] Lilly. Trulicity Cost Information. [WWW document]. URL https://pricinginfo.lilly.com/trulicity 2025.

[17] Lilly. Lilly to offer all approved doses of Zepbound (tirzepatide) single-dose vials through LillyDirect Self Pay Pharmacy Solutions. [WWW document]. URL https://investor.lilly.com/news-releases/news-release-details/lilly-offer-all-approved-doses-zepbound-tirzepatide-single-dose 2025.

[18] N. Nordisk. Novo Care. [WWW document]. URL https://www.novocare.com/obesity/products/wegovy/get-product.html 2025.

[19] Walker AM. Confounding by indication. Epidemiology. 1996;7:335–6. 8793355

[20] Mathur V, Wasden K, Shin TH, Medhati P, Nimeri AA, Tavakkoli A, et al. Neoadjuvant semaglutide, bariatric surgery weight loss, and overall outcomes. JAMA Surg. 2025;160:594–6. 10.1001/jamasurg.2025.0001 PMC11883598 40042864

[21] Çalık Başaran N, Dotan I, Dicker D. Post metabolic bariatric surgery weight regain: the importance of GLP-1 levels. Int J Obes 2024;49:412–17. 10.1038/s41366-024-01461-2 PMC11971041 38225284

