# Supplementary material for: Utilization and outcomes of nutrient-stimulated hormone therapies in patients undergoing bariatric surgery
Source: Int J Obes (Lond). 2026 Jul 20;50(10):2164–71. doi: 10.1038/s41366-026-02159-3 (PMC13722591; doi:10.1038/s41366-026-02159-3)
Supplement: Supplementary file 1 [file 41366_2026_2159_MOESM1_ESM.pdf]

Supplemental Table 1. Additional baseline characteristics of the study population by sleeve gastrectomy (SG) or Roux-en-Y gastric bypass (RYGB) and treatment groups

					SG			RYGB		
Characteristic	Total N=1638	SG N=1461 (89%)	RYGB N=177 (11%)	p-value[1]	NuSH N=520 (36%)	Non-NuSH N=941 (64%)	p-value[1]	NuSH N=36 (20%)	Non-NuSH N=141 (71%)	p-value[1]
Baseline height, m	1.67 ± 0.09	1.68 ± 0.09	1.66 ± 0.09	0.03	1.67 ± 0.10	1.68 ± 0.09	0.02	1.65 ± 0.08	1.66 ± 0.09	0.38
Baseline weight at time of surgery, kg	130.1 ± 24.6	130.9 ± 24.6	123.3 ± 23.5	0.0001	128.2 ± 23.8	132.4 ± 25.0	0.002	129.7 ± 21.6	121.7 ± 23.7	0.07
Baseline weight at first NuSH prescription, kg	103.8 ± 24.3	105.3 ± 24.2	91.3 ± 21.5	<0.0001	106.7 ± 23.1	104.5 ± 24.8	0.11	104.7 ± 19.2	87.8 ± 20.7	<0.0001
Comorbidities, n (%)										
Hypertension	924 (56)	831 (57)	93 (53)	0.27	335 (64)	496 (53)	<0.0001	23 (64)	70 (50)	0.13
Blood pressure, mmHg										
Systolic	131 ± 14	131 ± 14	130 ± 13	0.33	131 ± 15	132 ± 14	0.44	133 ± 12	130 ± 13	0.17
Diastolic	72 ± 10	72 ± 10	72 ± 10	0.56	71 ± 11	73 ± 10	0.004	73 ± 10	72 ± 10	0.79
HB A1C, %	6.0 ± 1.1	6.0 ± 1.1	5.9 ± 1.0	0.06	6.3 ± 1.3	5.9 ± 1.0	<0.0001	6.3 ± 1.2	5.8 ± 0.8	0.04
ALT, mg/dl	38 ± 25	38 ± 25	38 ± 27	0.85	39 ± 26	37 ± 25	0.19	35 ± 16	39 ± 29	0.31
AST, mg/dl	31 ± 17	30 ± 17	31 ± 17	0.77	32 ± 20	30 ± 15	0.03	30 ± 11	31 ± 18	0.67

Characteristic	Total N=1638	SG N=1461 (89%)	RYGB N=177 (11%)	p-value[1]	SG: NuSH N=520 (36%)	SG: Non-NuSH N=941 (64%)	p-value[1]	RYGB: NuSH N=36 (20%)	RYGB: Non-NuSH N=141 (71%)	p-value[1]
Lipids, mg/dl										
Total cholesterol[2]	173 ± 38	173 ± 39	177 ± 37	0.49	173 ± 41	173 ± 36	0.95	171 ± 42	179 ± 36	0.47
Triglycerides[2]	131(93, 186)	130(92, 184)	146(102, 194)	0.30	128(88, 191)	134 (96, 180)	0.52	159(98, 222)	135(102, 194)	0.75
HDL[2]	45 ± 12	45 ± 12	47 ± 10	0.35	46 ± 13	45 ± 11	0.62	44 ± 10	48 ± 10	0.16
LDL[2]	98 ± 32	98 ± 32	98 ± 35	0.93	98 ± 33	98 ± 31	0.93	93 ± 39	100 ± 33	0.54
Neighborhood indicators[3]										
Median income, ($)	87,280 ± 33,276	87,422 ± 33,484	86,109 ± 31,580	0.63	88,557 ± 33,909	86,779 ± 33,244	0.35	89,761 ± 36,007	85,196 ± 30,457	0.46
Percent unemployment	4.5 (2.5, 7.2)	4.4 (2.5, 7.3)	4.7 (2.5, 6.6)	0.32	4.3 (2.4, 7.3)	4.4 (2.5, 7.2)	0.27	3.4 (1.5, 6.4)	4.7 (2.7, 6.7)	0.08
Percent receiving SNAP benefits	7.2 (3.7, 14.7)	7.2 (3.7, 14.6)	7.4 (3.4, 15.6)	0.93	6.9 (3.6, 13.8)	7.5 (3.7, 15.0)	0.93	7.2 (3.7, 15.3)	7.5 (3.4, 15.6)	0.61
Percent education level										
Less than High School Diploma	5.6 (3.2,9.4)	5.6 (3.2,9.4)	5.8 (3.2,10.4)	0.43	5.3 (2.9,9.4)	5.8 (3.5,9.4)	0.67	5.1 (3.2,9.1)	5.9 (3.2,10.4)	0.87
High School and/or Some College	61.4(48.2, 70.4)	61.1(48.0, 70.0)	65.6(51.3, 72.3)	0.003	59.5(45.7, 68.3)	61.8(48.9, 70.5)	0.03	63.2(46.1, 74.5)	65.8(55.8, 71.9)	0.30

Characteristic	Total N=1638	SG N=1461 (89%)	RYGB N=177 (11%)	p-value[1]	SG: NuSH N=520 (36%)	SG: Non-NuSH N=941 (64%)	SG: p-value[1]	RYGB: NuSH N=36 (20%)	RYGB: Non-NuSH N=141 (71%)	RYGB: p-value[1]
Bachelor's Degree or Higher	30.5(20.5, 47.9)	31.1(20.9, 48.0)	27.2(18.0, 42.0)	0.007	33.6(22.9, 49.2)	30.5(20.3, 47.6)	0.05	29.5(14.4, 50.0)	27.2(19.2, 38.8)	0.41

Footnotes to supplemental table 1

Continuous data reported as mean ± SD or median (IQR).

[1] p-value from c^2 tests for categorical variables and t-test for continuous variables.

[2] N less than 1638 due to missing. Total cholesterol *n=565, TG *n=564, HDL n=570, LDL n=562.

[3] Obtained from geocoded data based zip code.

Supplemental Table 2. Description of nutrient stimulating hormone (NuSH) therapy prescription practices after bariatric surgery

	N	Average Months to Prescription	Average Length of Prescription Period (Months)	Average Number of Prescription Periods	Average Cumulative Months of Exposure
Overall	556	29.5 ± 21.9	5.6 ± 4.8	3.9 ± 3.3	20.2 ± 19.2
Sleeve Gastrectomy	520	29.9 ± 22.2	5.6 ± 4.9	3.9 ± 3.3	20.2 ± 19.2
No Diabetes	287	32.3 ± 21.5	5.4 ± 4.9	3.1 ± 2.4	15.7 ± 14.7
Diabetes	233	26.9 ± 22.8	5.9 ± 4.8	4.9 ± 3.9	25.7 ± 22.4
Roux-en-Y gastric bypass	36	22.8 ± 14.6	5.1 ± 3.0	4.0 ± 4.0	19.7 ± 20.4
No Diabetes	23	26.4 ± 14.1	4.7 ± 3.4	2.9 ± 2.4	12.8 ± 11.0
Diabetes	13	16.1 ± 13.9	5.7 ± 2.0	5.8 ± 5.5	31.9 ± 27.2

Supplemental table 3. Adjusted[1] average %TWL[2] (95% CI) from time of first nutrient stimulating hormone (NuSH) therapy prescription by prescription timing

NuSH therapy prescription timing	12 mo	24 mo	36 mo	42 mo	45 mo
Never	-1.5 (-2.5, -0.6)	-3.3 (-4.4, -2.2)	-4.8 (-6, -3.5)	-4.8 (-6.5, -3.2)	-5.8 (-7.7, -3.9)
Before Surgery	-2.3 (-8, 3.4)	-5.8 (-13.5, 1.9)	—	—	—
Before and After Surgery	2.9 (0.7, 5.0)	2.9 (0.4, 5.4)	4.6 (1.5, 7.7)	3.9 (-0.1, 7.8)	4.6 (0.2, 9.0)
After Surgery	4.5 (3.3, 5.6)	2.8 (1.5, 4.0)	1.1 (-0.4, 2.6)	1.1 (-0.6, 2.9)	2.2 (0.2, 4.2)

Footnotes to supplemental table 3.

[1]Model was adjusted for age, sex, BMI at first prescription, diabetes status, baseline hemoglobin A1C, type of surgery, hypertension, and percent of persons in zip code with high school diploma and/or some college.

[2]Estimated from least squared means of the interaction term between treatment and time from adjusted model.

Data were truncated at 45 months due to paucity of follow up information after that time point.

Supplemental table 4. Adjusted[1] average %TWL[2] (95% CI) from time of surgery by timing of nutrient stimulating hormone (NuSH) therapy

NuSH therapy prescription timing	12 mo	24 mo	36 mo	48 mo	60 mo	72 mo
Never	26.4 (25.5, 27.4)	25.6 (24.6, 26.5)	23.3 (22.2, 24.3)	22.5 (21.4, 23.6)	21.2 (19.9, 22.4)	19.6 (18.1, 21.2)
Before Surgery	22.9 (20.1, 25.8)	23.3 (20.0, 26.6)	19.2 (14.7, 23.7)	18.1 (10.6, 25.6)	16.5 (6.9, 26.1)	-
Before and After Surgery	21.1 (18.8, 23.4)	18.6 (16.2, 21.0)	19.6 (17.0, 22.1)	19.1 (16.3, 22.0)	19.0 (15.7, 22.3)	22.7 (17.9, 27.5)
After Surgery	22.5 (21.3, 23.6)	21.4 (20.2, 22.6)	20.0 (18.7, 21.2)	19.6 (18.3, 20.9)	19.1 (17.7, 20.5)	19.9 (18.3, 21.4)

Footnotes to supplemental table 4.

[1]Model was adjusted for age, sex, BMI at baseline, diabetes status, baseline hemoglobin A1C, type of surgery, hypertension, and percent of persons in zip code with high school diploma and/or some college.

[2]Estimated from least squared means of the interaction term between treatment and time from adjusted model.

Supplemental table 5. Adjusted[1] average %TWL[2] (95% CI) from time of surgery by first prescription time interval[3] for those who received a nutrient stimulating hormone (NuSH) therapy prescription after surgery[4]

NuSH therapy prescription category	12 mo	24 mo	36 mo	48 mo	60 mo	72 mo
0-6 months	21.9 (17.7, 26.0)	21.6 (17.2, 26.0)	23.1 (18.4, 27.9)	23.4 (17.9, 28.8)	25.1 (18.7, 31.4)	23.5 (16.1, 30.8)
6-12 months	16.7 (14.2, 19.2)	17.2 (14.7, 19.8)	16.2 (13.5, 18.9)	16.2 (13.2, 19.2)	16.7 (13.1, 20.4)	14.7 (10.1, 19.4)
12-24 months	19.3 (17.0, 21.6)	18.1 (15.8, 20.4)	18.9 (16.5, 21.3)	16.2 (13.6, 18.9)	15.9 (12.9, 18.9)	17.1 (13.6, 20.5)
24+ months	24.1 (22.3, 26.0)	21.9 (20.0, 23.8)	19.7 (17.8, 21.6)	19.8 (17.9, 21.7)	18.9 (17.0, 20.8)	20.2 (18.1, 22.3)

Footnotes to supplemental table 5.

[1]Model was adjusted for age, sex, BMI at baseline, diabetes status, baseline hemoglobin A1C, type of surgery, hypertension, and percent of persons in zip code with high school diploma and/or some college.

[2]Estimated from least squared means of the interaction term between treatment and time from adjusted model.

[3]Time intervals indicate the period in which patients received their first NuSH therapy prescription after surgery.

[4]Includes only patients who received a NuSH therapy prescription before and after surgery or only after surgery.
